# The value of five scoring systems in predicting the prognosis of patients with sepsis-associated acute respiratory failure

**DOI:** 10.1038/s41598-024-55257-5

**Published:** 2024-02-27

**Authors:** Shiqin Fan, Jing Ma

**Affiliations:** Department of Intensive Care Medicine, Liyuan Hospital, Tongji Medical College of Huazhong University of Science and Technology, Wuhan, Hubei China

**Keywords:** APSIII, LODS, OASIS, SOFA, SAPS II, Sepsis-related respiratory failure, Clinical trials, Respiratory distress syndrome

## Abstract

Our study aimed to identify the optimal scoring system for predicting the prognosis of patients with sepsis-associated acute respiratory failure (SA-ARF). All data were taken from the fourth version of the Markets in Intensive Care Medicine (MIMIC-IV) database. Independent risk factors for death in hospitals were confirmed by regression analysis. The predictive value of the five scoring systems was evaluated by receiving operating characteristic (ROC) curves. Kaplan‒Meier curves showed the impact of acute physiology score III (APSIII) on survival and prognosis in patients with SA-ARF. Decision curve analysis (DCA) identified a scoring system with the highest net clinical benefit. ROC curve analysis showed that APS III (AUC: 0.755, 95% Cl 0.714–0.768) and Logical Organ Dysfunction System (LODS) (AUC: 0.731, 95% Cl 0.717–0.7745) were better than Simplified Acute Physiology Score II (SAPS II) (AUC: 0.727, 95% CI 0.713–0.741), Oxford Acute Severity of Illness Score (OASIS) (AUC: 0.706, 95% CI 0.691–0.720) and Sequential Organ Failure Assessment (SOFA) (AUC: 0.606, 95% CI 0.590–0.621) in assessing in-hospital mortality. Kaplan‒Meier survival analysis patients in the high-APS III score group had a considerably poorer median survival time. The DCA curve showed that APS III may provide better clinical benefits for patients. We demonstrated that the APS III score is an excellent predictor of in-hospital mortality.

## Introduction

Sepsis is a severe life-threatening organ dysfunction caused by a dysregulated host response to infection and is associated with a high risk of mortality^1^. Acute respiratory failure (ARF) is a common complication of sepsis and may lead to severe pulmonary organ damage, causing serious illness or even death^2^. Through a series of mechanisms, sepsis induces the expansion of a systemic inflammatory response that can cause lung damage and severe respiratory distress, leading to ARF^3,4^. When SA-ARF develops, the prognosis is bleak, resulting in critical death^5,6^. An increase in mortality is associated with a higher treatment expense. According to an epidemiological report from the United States, the cost of treating respiratory failure might reach $5 billion per year^7^. Early assessment and prediction of disease prognosis, as well as prompt management, are critical for reducing mortality and high health care expenditures in hospitalized patients^8^. However, studies on the prognostic prediction of patients with SA-ARF are rare^9^. An optimal scoring system is needed to create accurate prognostic predictions for patients.

Scoring systems are routinely used in the ICU to evaluate the prognosis of disease^10^. The SOFA score was created in 1996^11^ and is included in the current definition of sepsis^12^. The SOFA score is valuable for predicting the prognosis of sepsis patients^13–15^. The APS III score is a component of the Acute Physiology and Chronic Health Evaluation II (APACHE II), which is simpler than APACHEII. It lacks an age score or a chronic health score, and is more accessible for clinical use than the APACHE II score^16^. APSIII can predict mortality in the ICU^17^. The SAPS II was created in 1993, it was used to predict the risk of death in ICU^18^. The SAPS II has been widely used to predict the prognosis in a variety of diseases, including acute coronary syndrome (ACS)^19,20^. The LODS is an objective tool proposed in 1996, which has been used to assess the severity of organ dysfunction in ICU^14^. The LODS can also predict prognosis in patients with sepsis or ARF^21,22^. In 2013, Johnson et al. reported OASIS, a predictive scoring system built with machine learning methods. The OASIS helps to predict mortality in patients admitted to the ICU^23^. It is also a good predictor of prognosis in sepsis and respiratory disease^24^, and it can be used to predict mortality in the ICU^25^. The purpose of this study is to compare which is among those severity scores the one that better predicts mortality in hospital with SA-ARF.

## Methods

### Data source

All data were obtained from a large-scale, publicly available database, the MIMIC-IV database, developed by the Massachusetts Institute of Technology. The MIMIC-IV database includes data from all patients treated in the ICU at Beth Israel Deaconess Medical Center between 2008 and 2019, including information on admission examination, laboratory tests, therapeutic measures, etc. Researchers were required to pass the relevant tests on the on the National Institute of Health website and sign a statement to ultimately qualify for access to the database^26^. The author, Fan Shiqin, finished the online training course for data research (record ID: 52287583) and signed the agreement. The relevant Institutional Review Board in America approved the project. In addition, the program is compliant with the Health Insurance Portability and Accountability Act (HIPPA). The identifying information about the patients was removed and we were not required to obtain informed consent or ethical review.

### Study population

All adult patients diagnosed with SA-ARF (first admission only) were extracted from the MIMIC-IV database. Inclusion criteria: (1) meets the criteria for the diagnosis of sepsis 3.0^12^, patients with probable infection and SOFA score ≥ 2; (2) ARF determined using the International Classification of Diseases (icd_codes): "51,881", "J9602", "51,851", "J9601", "J9600"; (3) re-admitted patients, with only the diagnosis from the first admission was retained; (4)) admitted to ICU and age ≥ 18 years; (5) admission time > 1 day; exclusion criteria: (1) SOFA < 2; (2) missing values from the baseline data were excluded, such as PaO_2_/FiO_2_, lactate, Hb, INR, WBC, BUN, BP, T, and RR were not recorded.

### Data extraction and management

We used Navicat Premium software (version 15.0) to extract relevant data from the MIMIC-IV database. MIMIC-IV provided all of the needed data, which included APSIII, SAPSII, OASIS, LODS, and SOFA scores. We also included covariates that might influence the relationship between these five scoring systems and in-hospital mortality, extracting the following basic data: age, sex, race, and whether the patients were mechanically ventilated. The laboratory parameters included hematocrit (HCT), platelet (PLT), hemoglobin (HB), white blood cell (WBC), prothrombin time (PT), creatinine (Cr), blood urea nitrogen (BUN), the international normalized ratio (INR), lactate, the ratio of partial pressure of O_2_ in arterial blood to the fraction of inspired oxygen (PaO_2_/FiO_2_), vital signs, such as heart rate (HR), mean arterial pressure (MBP), the respiratory rate (RR) and temperature (T). The comorbidities included hypertension, liver disease, renal disease, chronic pulmonary disease (CPD), and diabetes. If a laboratory test or vital sign was measured more than once on the first day of hospitalization, the median value was obtained.

### Statistical analysis

We used R (version 4.2.2), SPSS (version 27) and MedCalc (version 20.0.22) to analyze the data. Normally distributed data were expressed as mean ± standard deviation using the independent samples t-test; non-normally distributed data were expressed as median (interquartile spacing) [M (QL, QU)] using the Mann–Whitney test. Categorical variables were analyzed using t-test or chi-square test and expressed as numbers and percentages. Regression analyses were performed to identify independent risk factors for death in the hospital. Variables with P < 0.05 in the univariate analysis were incorporated into the multivariate analysis. The AUC of the ROC curves were compared using the method of Delong et al.^27^ to determine the predictive ability of each scoring system for hospital death. After the optimal cutoff value was obtained from the ROC curve, Kaplan–Meier curves were plotted to assess the impact of APSIII on the survival prognosis of patients in the high and low subgroups. The log rank test was used to evaluate the difference in survival between the high-stage and low-stage patients. Finally, DCA was applied to assess the net benefits of the five scoring systems for patients with SA-ARF^28^. The net benefit is a decision analysis metric that combines the assessment of harms and benefits. It is a clinical judgment of the relative value of the benefits and harms of a certain examination. A series of net benefits can be obtained by plotting via a DCA, which can be used to assess diagnosis and prognosis^29^. The larger the area under the DCA curve is, the greater the clinical benefit of a scoring system will be.

## Results

### Baseline characteristics

**Figure 1:**
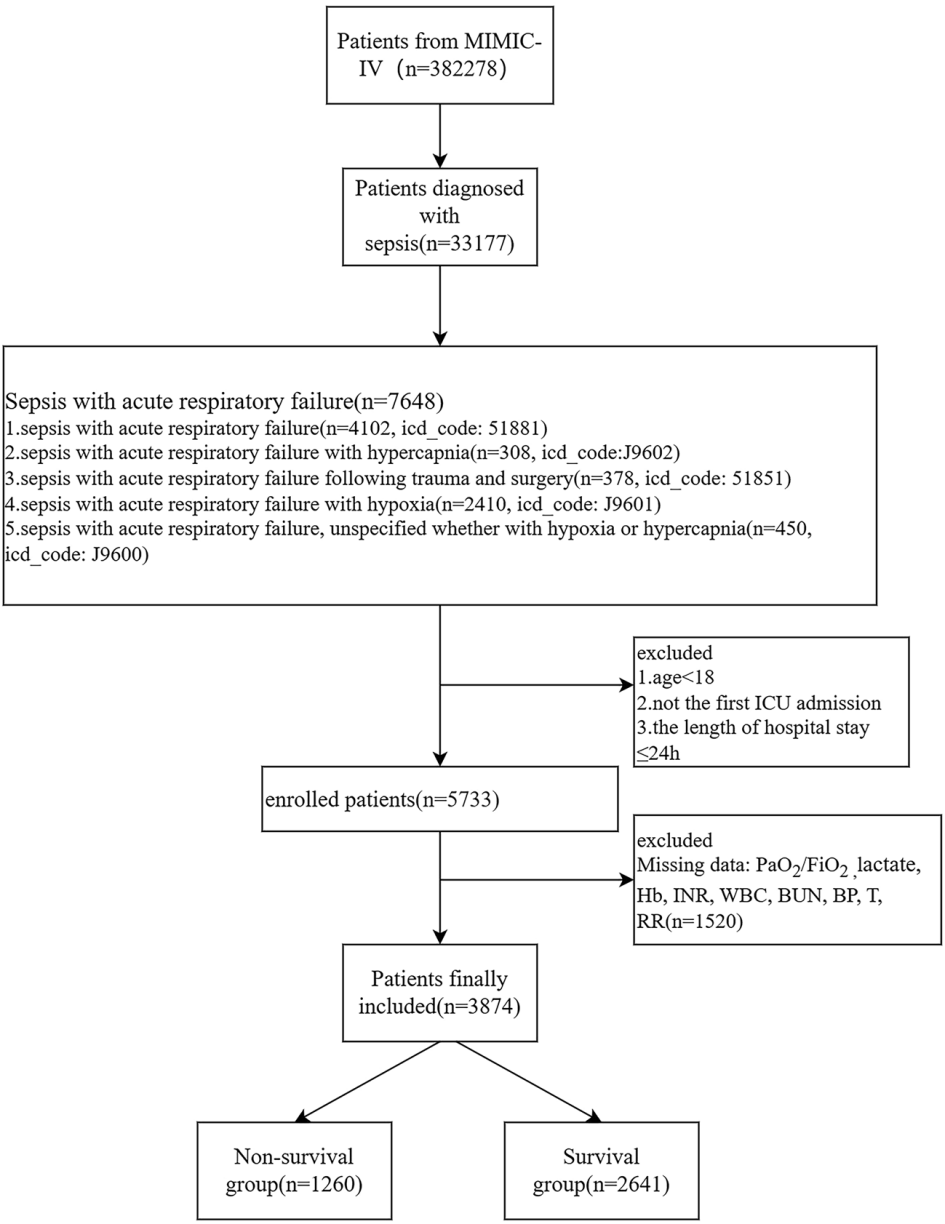
Flow chart of this study. *PaO*_*2*_*/FiO*_*2*_ the ratio of partial pressure of O_2_ in arterial blood to the fraction of inspired oxygen; *Hb* haemoglobin, *INR* the international normalized ratio, *WBC* white blood cell, *BUN* blood urea nitrogen, *BP* blood pressure, *T* temperature, *RR* the respiratory rate.

We extracted data on 7648 patients with SA-ARF from the MIMIC-IV database. After further screening and elimination, 3874 eligible patients were included. We divided them into 1260 non-survival and 2641 survival groups. The flow chart of the data extraction process is available in Fig. 1. The average age of the survival patients was 65 years, and the non-survival group was 67 years. The average age was lower in the survivor group than in the non-survival group (P < 0.05). The length of hospital stay (LOS Hos) and length of ICU stay (LOS ICU) were longer in the survival group than in the non-survival group (P < 0.001). Among the laboratory results on the day of admission, the WBC, Cr, BUN, INR, PT, lactate, HR, and RR were significantly higher in the non-survival group than the survival group (P < 0.05). A higher PLT levels, Hb, T, BP, and MAP were found in the survival group than in the non-survival group (P < 0.001). The non-survival group was more likely to have the following coexisting comorbidities than was the survivor group: renal disease (P < 0.05). All five scoring systems were high in the non-survival group compared to the survival group. Information related to sex, age, laboratory test results, and the scoring systems used for patients with SA-ARF is shown in Table 1.

**Table 1:** Demographic and clinical characteristics of patients (admission variable only).

	Total	Non-survival	Survival	P
Patients, n (%)	3874	1260 (32.5%)	2614 (67.5%)	
Demographics				
Age (years)	66 (55, 78)	67 (56, 79)	65 (52, 76)	< 0.001
Male (%)	2244 (57.9%)	725 (57.5%)	1519 (57.5%)	0.736
LOS Hos (days)	11.4 (6, 19.1)	10.5 (4.9, 17.7)	13.6 (8.2, 21.5)	< 0.001
LOS ICU (days)	6.0 (2.9, 11.2)	6 (3, 11)	6.4 (3.36, 12)	< 0.001
White (%)	2280 (58.8%)	705 (56.0%)	1575 (59.6%)	0.011
Life-support				
Mechanical ventilation (%)	3084 (79.5%)	1004 (79.7%)	2076 (79.4%)	0.056
Laboratory tests				
Hb (g/dl)	9.9 (8.4, 11.6)	9.8 (8.3, 11.5)	10.1 (8.5, 11.7)	< 0.001
WBC (10^9^/L)	10.4 (7.1, 14.4)	10.6 (7.1, 14.7)	10.2 (7.2, 13.8)	0.014
PLT (10^9^/L)	165 (106, 231)	162 (101, 228)	174 (118, 236)	< 0.001
BUN (mmol/L)	27 (18, 46)	28 (18, 47)	24 (16, 40)	< 0.001
Cr (mg/dl)	1.2 (0.7, 1.7)	1.1 (0.7, 1.8)	1.0 (0.7, 1.5)	< 0.001
INR	1.2 (1.1, 1.5)	1.2 (1.1, 1.5)	1.2 (1.1, 1.4)	< 0.001
PT(s)	13.6 (12.2, 16.2)	13.8 (12.2, 16.8)	13.3 (12, 15.3)	< 0.001
Lactate (mmol/L)	1.5 (1.1, 2.1)	1.5 (1.1, 2.3)	1.3 (1.0, 1.9)	< 0.001
PaO_2_/FiO_2_ (mmHg)	117.5 (74.0, 205.0)	113 (70, 200)	125 (80, 241)	< 0.001
PaO_2_/FiO_2_ < 300 mmHg	3427 (88.4%)	1153 (91.5%)	2274 (87.0%)	< 0.001
Vital signs				
T (℃)	37.0 (36, 37.4)	37.0 (36.6, 37.4)	37.1 (36.7, 37.5)	< 0.001
HR (bmp)	88.9 (88.9, 101.8)	89.6 (77.3, 103.0)	87.4 (76.3, 99.6)	< 0.001
RR (cmp)	20.4 (17.8, 23.6)	20.9 (18.0, 24)	20 (17.5, 22.9)	< 0.001
MAP (mmHg)	75.1 (69.6, 82.1)	75 (69, 82)	76 (70, 82.9)	< 0.001
Coexisting comorbidities				
Hypertension	1475 (38.0%)	438 (34.8%)	1037 (39.2%)	0.003
Chronic pulmonary disease	1190 (30.7%)	378 (30%)	812 (31.0%)	0.501
Liver disease	720 (18.5%)	227 (18.0%)	493 (18.7%)	0.527
Renal disease	815 (21.0%)	295 (23.4%)	520 (19.9%)	0.012
Diabetes	1134 (29.2%)	368 (29.2%)	766 (29.0%)	0.950
Scoring systems				
APSIII	68 (49, 92)	73 (52, 97)	66 (44, 80)	< 0.001
SAPSII	45 (35, 57)	55 (44, 67)	42 (33, 51)	< 0.001
OASIS	42 (36, 48)	43 (37, 49)	40 (34, 46)	< 0.001
LODS	8 (6, 11)	9 (6, 11)	7 (5, 10)	< 0.001
SOFA	4 (2, 5)	4 (2, 6)	3 (2, 5)	< 0.001

*Hb* Hemoglobin, *WBC* White blood cell, *PLT* Blood platelet, *BUN* Blood urea nitrogen, *Cr* Creatinine, *INR* International normalized ratio, *PT* Prothrombin time, *PaO*_*2*_ Arterial oxygen pressure, *FiO*_*2*_ Inspired oxygen fraction, *T* Temperature, *HR* Heart rate, *RR* Respiratory rate, *MAP* Mean arterial pressure, *APSIII* acute physiology score III, *SAPSII* Simplified acute physiology score, OASIS Oxford acute severity of illness score, *LODS* Logistic organ dysfunction system, *SOFA* Sequential organ failure assessment.

### Regression analysis

#### Logistic regression analysis

Multivariate analysis showed that age (OR: 1.031, 95% CI 1.025–1.037, P < 0.001), RR (OR: 1.030, 95% CI 1.009–1.051, P = 0.004), lactate (OR: 1.385, 95% CI 1.300–1.476, P < 0.05), APSIII (OR: 1.021, 95% CI 1.015–1.026, P < 0.001), LODS (OR: 1.079, 95% CI 1.037–1.122, P < 0.001), and PaO_2_/FiO_2_ (OR: 1.001, 95% CI 1.000–1.002, P = 0.036) (Table 2) were independent risk factors for death in hospitals in patients with SA-ARF. In contrast, hypertension (OR: 0.752, 95% CI 0.627–0.901, P < 0.05) and renal disease (OR: 0.751, 95% CI 0.608–0.929, P = 0.008) were protective factors.

**Table 2:** Binomial logistic regression analysis of in-hospital mortality in patients with SA-ARF in ICU.

Variable	Univariate analysis		Multivariate analysis	
	P	OR (95%Cl)	P	OR (95% Cl)
Age (years)	< 0.001	1.022 (1.017, 1.026)	< 0.001	1.029 (1.023, 1.035)
Male	0.736	0.977 (0.853, 1.119)		
APSIII	< 0.001	1.035 (1.032, 1.038)	< 0.001	1.020 (1.015, 1.026)
OASIS	< 0.001	1.095 (1.083, 1.106)	0.927	1.001 (0.985, 1.016)
LODS	< 0.001	1.283 (1.251, 1.317)	0.001	1.069 (1.026, 1.114)
SOFA	< 0.001	1.164 (1.127, 1.202)	0.525	0.989 (0.955, 1.024)
SAPSII	< 0.001	1.055 (1.049, 1.060)	0.129	1.006 (0.998, 1.013)
RR	< 0.001	1.100 (1.083, 1.118)	0.005	1.030 (1.009, 1.051)
Mechanical ventilation	0.056	0.831 (0.687, 1.005)	0.424	0.897 (0.686, 1.172)
Hypertension	0.003	0.801 (0.704, 0.932)	0.002	0.751 (0.627, 0.901)
Pulmonary disease	0.501	0.951 (0.822, 1.101)		
Renal disease	0.012	1.231 (1.047, 1.447)	0.007	0.747 (0.604, 0.923)
Lactate(mmol/L)	< 0.001	1.663 (1.567, 1.765)	< 0.001	1.375 (1.290, 1.466)
PaO_2_/FiO_2_(mmHg)	< 0.001	0.998 (0.997, 0.999)	0.031	1.001 (1.000, 1.002)
PaO_2_/FiO_2_ < 300 mmHg	< 0.001	1.611 (1.282, 2.025)	0.100	1.394 (0.939, 2.069)

*PaO*_*2*_ Arterial oxygen pressure, *FiO*_*2*_ Inspired oxygen fraction, *RR* Respiratory rate, *APSIII* Acute physiology score III, *SAPSII* Simplified acute physiology score, *OASIS* Oxford acute severity of illness score, *LODS* Logistic organ dysfunction system, *SOFA* Sequential organ failure assessment.

#### Cox regression analysis

Independent risk factors for death were obtained using Cox regression analysis. Multivariate analysis revealed that APS III (HR: 1.005, 95% CI 1.002–1.009, P = 0.004), LODS (HR: 1.030, 95% CI 1.002–1.059, P = 0.037), SAPS II (HR: 1.007, 95% CI 1.002–1.013, P < 0.001), RR (HR: 1.034, 95% CI 1.020–1.049, P < 0.001) and lactate (HR: 1.166, 95% CI 1.139–1.193, P < 0.001) (Table 3) were found to be independent predictors of in-hospital death for patients with SA-ARF.

**Table 3:** Cox regression analysis of risk factors for in-hospital death in patients with SA-ARF in ICU.

Variable	Univariate analysis		Multivariate analysis	
	P	HR (95% Cl)	P	HR (95% Cl)
Age (years)	0.002	0.994 (0.991, 0.998)	< 0.001	1.020 (1.015, 1.024
Male	0.335	1.057 (0.945, 1.181)		
APSIII	< 0.001	1.016 (1.014, 1.017)	< 0.001	1.012 (1.008, 1.016)
OASIS	< 0.001	1.045 (1.038, 1.052)	0.635	1.003 (0.992, 1.014)
LODS	< 0.001	1.121 (1.103, 1.139)	0.025	1.034 (1.004, 1.065)
SOFA	< 0.001	1.080 (1.060, 1.101)	0.776	1.003 (0.982, 1.024)
SAPSII	< 0.001	1.016 (1.014, 1.017)	0.004	1.007 (1.002, 1.013)
RR	< 0.001	1.100 (1.083, 1.118)	< 0.001	1.034 (1.019, 1.048)
Mechanical ventilation	< 0.001	1.371 (1.168, 1.608)	0.194	0.877 (0.720, 1.069)
Hypertension	0.128	0.914 (0.814, 1.026)		
Pulmonary disease	< 0.001	0.809 (0.717, 0.913)	0.411	1.052 (0.932, 1.189)
Renal disease	< 0.001	1.073 (1.061, 1.086)	0.464	1.052 (0.919, 1.205)
Lactate(mmol/L)	< 0.001	1.232 (1.209, 1.256)	< 0.001	1.190 (1.163, 1.218)
PaO_2_/FiO_2_(mmHg)	< 0.001	0.999 (0.998, 0.999)	0.253	1.001 (1.000, 1.001)
PaO_2_/FiO_2_ < 300 mmHg	< 0.001	1.437 (1.178, 1.751)	0.294	0.851 (0.629, 1.151)

*PaO*_*2*_ Arterial oxygen pressure, *FiO*_*2*_ Inspired oxygen fraction, *RR* Respiratory rate, *APSIII* Acute physiology score III, *SAPSII* Simplified acute physiology score, *OASIS* Oxford acute severity of illness score, *LODS* Logistic organ dysfunction system, *SOFA* Sequential organ failure assessment.

### Comparison of Receiver operating characteristic curves for five scoring systems

**Figure 2:**
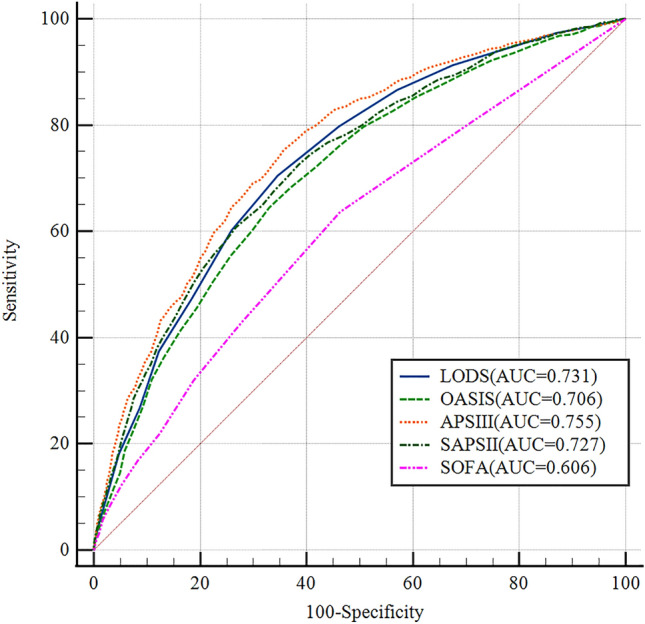
Receiver operating characteristic curves. (1) ROC curves of APSIII, SAPSII, LODS, OASIS and SOFA. (2) *APSIII* acute physiology score III, *SAPSII* Simplified acute physiology score, *OASIS* Oxford acute severity of illness score, *LODS* Logistic organ dysfunction system, *SOFA* Sequential organ failure assessment.

The predictive value of the scoring system for in-hospital mortality was compared using AUC. The ROC curves are shown in Fig. 2, APSIII (AUC: 0.755, 95% Cl 0.714–0.768), LODS (AUC: 0.731, 95% Cl 0.717–0.7745), SAPSII (AUC: 0.727, 95% CI 0.713–0.741), OASIS (AUC: 0.706, 95% CI 0.691–0.720) and SOFA (AUC: 0.606, 95% CI 0.590–0.621) (Table 4). The AUCs of LODS, OASIS, and APS III were more than 0.7, which were significantly higher than the AUC value of SOFA. The AUC of APS III was greater than that of LODS. APSIII had the highest AUC and was more reliable in predicting death in the hospital. The threshold of the scoring system corresponding to Youden's index was chosen as the best threshold for predicting death in the hospital. APSIII had the highest Youden’s index (0.367) and sensitivity (73.53%), its specificity (63.2%) was within tolerable bounds, and OASIS had the highest specificity (67.06%).

**Table 4:** Comparisons of different predictive index.

Factor	AUC	95%Cl	Optimal cut-off	Sensitivity	Specificity	Youden’s index	P-value	Z-value
APSIII	0.755	0.741–0.768	69	73.53	63.2	0.367	Ref	Ref
LODS	0.731	0.717–0.745	8	70.48	65.46	0.359	< 0.001	4.350
OASIS	0.706	0.691–0.720	43	64.44	67.06	0.315	< 0.001	6.715
SAPSII	0.727	0.713–0.741	45	72.54	61.51	0.340	< 0.001	3.346
SOFA	0.606	0.590–0.621	3	63.65	53.71	0.174	< 0.001	14.317

*APSIII* Acute physiology score III, *SAPSII* Simplified acute physiology score, *OASIS* Oxford acute severity of illness score, *LODS* Logistic organ dysfunction system, *SOFA* Sequential organ failure assessment, *AUC* area under the ROC curve, *Cl* confidence interval, *P-value/Z-value* compared with APSIII.

### Kaplan‒Meier curves of the APSIII scoring system

**Figure 3:**
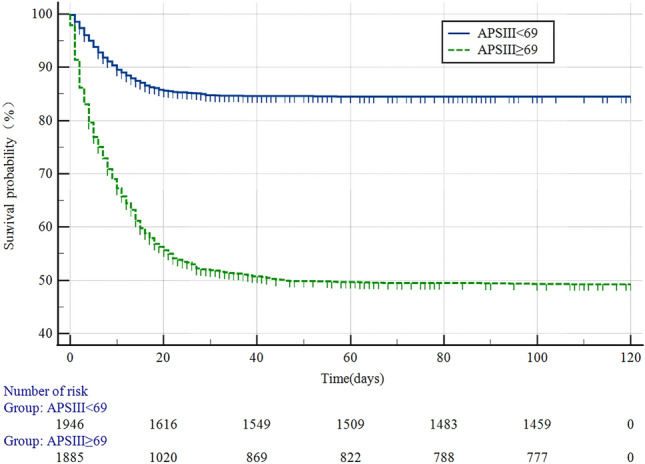
Kaplan–Meier curves for patients with SA-ARF with APSIII ≥ 69 and APSIII < 69. *APSIII* Acute physiology score III.

The ideal cutoff value for the APSIII score was 69 according to the ROC curve and Youden's index calculations, splitting the patients in the APSIII subgroup into two subgroups: high- and low-scoring subgroups (Fig. 3). In the low subgroup (APSIII < 69), the median survival was 102.824 days (95% CI 101.030–104.619), while in the high subgroup, it was 64.278 days (95% CI 61.773–66.783). There was a statistically significant difference in survival time between the high and low subgroups (χ^2^ = 539.6405, P < 0.001). The hazard ratio (HR) for the low subgroup compared to the high subgroup was 0.263 (95% Cl 0.235–0.294), indicating that the risk of death in the low subgroup was 0.263 times greater than that in the high subgroup. Therefore, the prognosis of the low subgroup was superior to that of the high subgroup.

### Comparison of decision curve analysis curves

**Figure 4:**
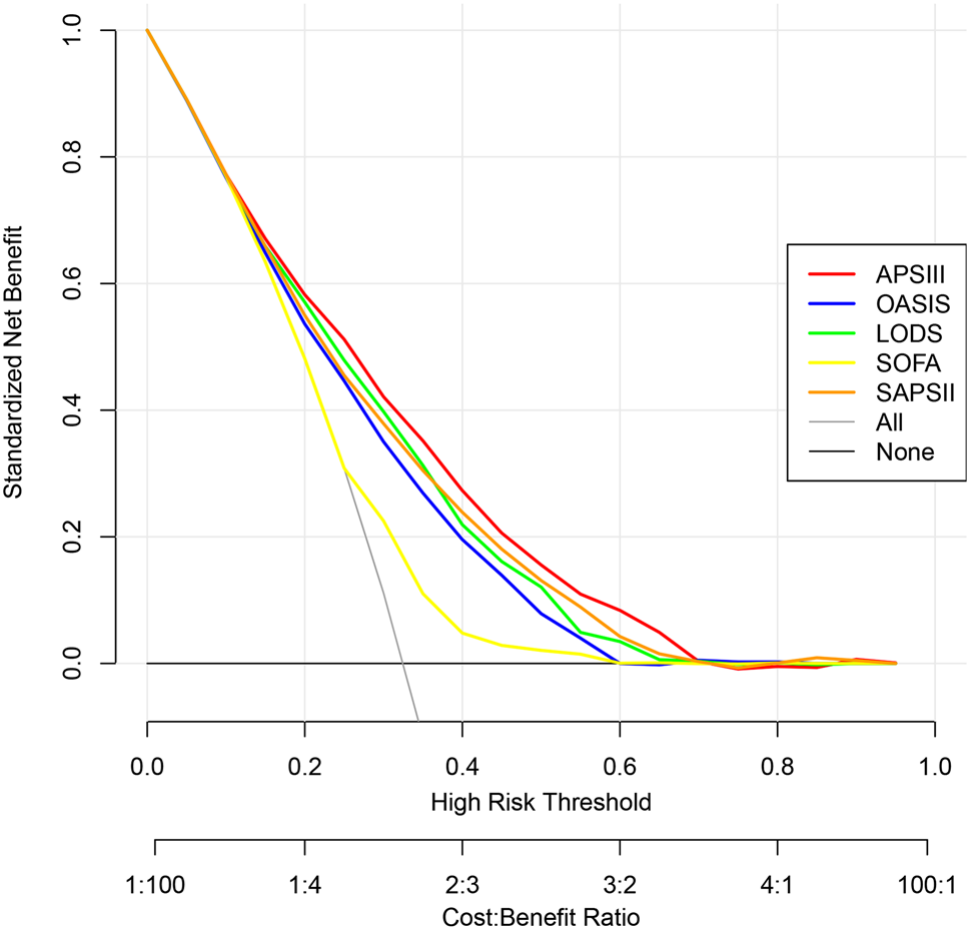
Comparison of decision curve analysis curves. The clinical benefit of the scoring system can be assessed by assessing the net benefit (y-axis) over a range of threshold probabilities (x-axis). The grey line represents the assumption that all patients with SA-ARF present with in-hospital death, and the black line indicates the assumption that no patients with SA-ARF present with in-hospital death. It can be concluded that the APSIII curve (red) shows the greatest benefit compared to the other curves. *APSIII* acute physiology score III, *SAPSII* Simplified acute physiology score, *OASIS* Oxford acute severity of illness score, *LODS* Logistic organ dysfunction system, *SOFA* Sequential organ failure assessment.

As shown in Fig. 4. The results of the DCA curve showed that the red line representing APSIII always above the other lines (LODS, SAPSII, OASIS, SOFA in descending order). Therefore, APS III has the best net benefit and provides the best clinical benefit. We can utilize the APS III score for timely clinical interventions to achieve better clinical benefits.

## Discussion

Early identification of patients with SA-ARF is important for prognosis. We can implement mechanical ventilation, infection control and various urgent interventional treatments as soon as possible, which will help reduce the risk of death. We aim to find a scoring system that will help us predict prognosis at an early clinical stage, so that physicians can intervene early in this group of high-risk patients to reduce in-hospital mortality. APSIII, SAPSII, LODS, OASIS, and SOFA are some typical scoring systems, and the prognostic value of these scoring systems in patients with SA-ARF is worth investigating.

We have come to the following conclusions: (1) both logistic regression and Cox regression results indicated that APSIII and LODS were independent risk factors for predicting in-hospital mortality in patients with SA-ARF. (2) ROC curves suggested that the AUCs of APSIII, SAPSII, LODS, and OASIS were above 0.7, which showed some clinical predictive value. APSIII had the best predictive value for assessing in-hospital mortality, while SOFA had the poorest performance. (3) In-hospital mortality was associated with LODS, OASIS, SOFA and APSIII scores, with higher scores representing more severe organ failure and worse prognosis. (4) Kaplan‒Meier survival curves showed that patients with APSIII values greater than 69 had lower median survival days and a higher in-hospital mortality rate. The log rank test of two survival curves were P(sig.) < 0.01, and the Breslow test were P(sig.) < 0.01, suggesting that there was a difference between the APSIII score and prognosis of patients. (5) DCA curves revealed that APS III had the greatest beneficial effect on patients within the maximum threshold, with timely clinical intervention may improve clinical benefit. The other four scoring systems, in descending order of clinical benefit, were LODS, SAPSII, OASIS, and SOFA.

In addition, several laboratory indicators, lactate levels, and the RR are also independent risk factors for in-hospital death in patients with SA-ARF. The release of metabolites and lactic acid increases, and causes respiratory acidosis^30^. Therefore, the lactate concentration can be used to evaluate the overall outcome of patients with sepsis^13^. When SA-ARF occurs, it may cause acidosis, which is negative for the patient's prognosis. Some indicators can be used to document the degree of acidosis, such as the RR and lactate level^31^. When sepsis develops to ARF, the body is deprived of oxygen due to impaired gas exchange, causing shortness of breath and a buildup of lactic acid. The accumulation of lactic acid is associated with mortality^32^. In addition, we discovered that age was an important risk factor associated with death in the hospital. This might be because the risk of death from sepsis increases with age^33^.

APSIII is a common scoring system in the ICU. APSIII is part of the acute physiological score of the APACHE II score, which contains 12 indicators, namely, T, MAP, HR, RR, PaO_2_, pulmonary artery oxygen differential (A-aDO_2_), hematocrit (HCT), serum potassium (K ^+^), serum sodium (Na ^+^), pH, Cr, and WBC count; it is simpler than the APACHE II score^16^. APSIII can be used in pediatric ICU, neurological ICU, and acute pancreatitis for determining prognosis and predict risk of death^34–36^. Fernández et al.^37^ found that the APSIII score was related to 90-day fatality in patients with sepsis-related acute kidney injury (Cox regression: hazard ratio (HR): 1.01, 95% confidence interval (Cl): 1.0–1.0, p < 0.048). We found that the APSIII had the highest sensitivity and Youden’s index, and it had the largest AUC of 0.755 (95% CI 0.741–0.768). The DCA curve also showed that the APSIII provide the best clinical benefit to patients. Kaplan‒Meier curves also revealed that higher APSIII scores were associated with poorer prognosis. The reason why APSIII has the best predictive value may be related to its indicators, pH and RR, which allow it to identify disturbances in acid‒base balance. As mentioned above, ARF can lead to respiratory acidosis through deepened and accelerated breathing and accumulate lactic acid in the body^31^. An imbalance in alkaline balance leads to disturbances in electrolyte metabolism, which can be reflected by indicators such as Na^+^ and K^+^ in APSIII. APSIII is also an indicator of lung ventilation. When ARF occurs, the prognosis is related to oxygen uptake in the lungs. When pulmonary ventilation is impaired, this can exacerbate the progression of the disease. This is an overlooked component of other scoring systems. There are no studies on the value of the APSIII scoring system for in-hospital mortality in patients with SA-ARF. We found that the APSIII score has the optimal predictive value.

Zhu et al.^38^ discovered in a study of scores in patients with sepsis that the AUC for SAPS II is (AUC: 0.754, 95% CI 0.743–0.765), for OASIS is (AUC: 0.753, 95% CI 0.742–0.764), and for LODS is (AUC: 0.822, 95% CI 95.0–743.0), which is comparable to our study that LODS (AUC: 0.731, 95% CI 0.717–0.745), OASIS (AUC: 0.706, 95% CI 0.691–0.720), and SAPSII (AUC: 0.727, 95% CI 0.713–0.741). The indicators of LODS involve several systems, including the neurological, circulatory, renal, respiratory, and hepatic systems^39^. In severe ARF, the PaO_2_/FiO_2_ ratio can be used to indicate pulmonary ventilation, but it may be biased when accompanied by increased PaCO_2_. Finally, the LODS score demonstrated good predictive value in this investigation. The regression analysis showed that it was an independent risk factor for death in the hospital, and the clinical benefit of the AUC and DCA curves was second only to that of the APS III score. SAPSII includes seventeen variables, including age, physiological variables, and chronic diseases, and it also contains indicators reflecting electrolyte disturbances such as Na^+^, k^+^, HCO_3_^−^ and PaO_2_/FiO_2_. However, it is not as accurate as APSIII for predicting SA-ARF, and its predictive value lies at an average level. As previously described^21^, OASIS is a machine learning algorithm-based model that does not have as many for determining organ failure as does APSIII. None of these three scoring systems has significant advantages over the APSIII. Moreover, these methods do not perform as well as APSIII on the DCA curve and have poorer clinical benefits than APSIII.

Notably, the SOFA score performed the worst in terms of predictive value. The SOFA score is a common indicator used to assess organ failure in patients with sepsis^40^. Ferreira et al.^41^ found that when SOFA score were assessed in the first 96 h in the ICU, the mortality increased with increasing SOFA score. Zeng et al.^42^ discovered that the SOFA score was related to mortality from ARF in patients with lung cancer (HR: 1.142, 95% CI 1.012–1.288, p = 0.031). However, both logistic and Cox regression analyses suggested that the SOFA score is not an independent risk factor for predicting death in the hospital. ROC curves also showed that it had the lowest AUC (AUC: 0.606, 95% CI 0.590–0.621). The DCA curve showed that the score was at the bottom of the curve. The SOFA score has the lowest predictive value and clinical benefit. It may be that it has considerably fewer indicators than a valuable scoring system for appeals.

The strength of our study is that our study is retrospective and based on the large database-MIMIC-IV. We studied a large sample size. The results of our study are also convincing. The data extracted from the database provides credibility to this study. The data extracted from the database lend credibility to this study.

This study has several limitations. First, our study was only a single-center retrospective analysis of the MIMIC-IV database, and we did not use external data to validate the conclusions. Second, the database we used has limitations. We strictly followed the ICD diagnostic codes to identify sepsis and ARF, and we ultimately obtained data on the SA-ARF. The database did not contain a specific classification of the site of infection, nor was it detailed enough to categorize the type of ARF; therefore, we were unable to perform subgroup analyses (e.g., whether it originated from pneumonia, hospital acquired pneumonia (HAP), or acute respiratory distress syndrome (ARDS)) to draw more convincing conclusions. Third, we were unable to determine whether in-hospital deaths were caused by SA-ARF or by the patient's ultimate decision to abandon treatment. Therefore, additional large clinical cohort studies are needed to validate the accuracy of these findings.

## Conclusion

APSIII and LODS are independent risk factors for mortality in patients who develop SA-ARF. ROC and DCA curves showed that APSIII had the best predictive value. APSIII is an excellent predictor of prognosis in patients with SA-ARF, and it can predict in-hospital mortality more accurately. Early assessment of a patient's risk of death allows physicians to make the best clinical decisions and helps to improve the prognosis of patients.

## Data Availability

Our data were extracted from the public databases, MIMIC-IV databases. The data in the MIMIC database are available from the https://mimic.physionet.org/.

## References

[1] O'Brien JM, Ali NA, Aberegg SK, Abraham E (2007). Sepsis. Am. J. Med..

[2] Cochi SE, Kempker JA, Annangi S, Kramer MR, Martin GS (2016). Mortality trends of acute respiratory distress syndrome in the United States from 1999 to 2013. Ann. Am. Thorac. Soc..

[3] Zhou X, Liao Y (2021). Gut-lung crosstalk in sepsis-induced acute lung injury. Front. Microbiol..

[4] Zampieri FG, Mazza B (2017). Mechanical ventilation in sepsis: A reappraisal. Shock.

[5] Lelubre C, Vincent JL (2018). Mechanisms and treatment of organ failure in sepsis. Nat. Rev. Nephrol..

[6] Fowler AA (2019). Effect of vitamin C infusion on organ failure and biomarkers of inflammation and vascular injury in patients with sepsis and severe acute respiratory failure: The CITRIS-ALI randomized clinical trial. JAMA.

[7] Stefan MS (2013). Epidemiology and outcomes of acute respiratory failure in the United States, 2001 to 2009: A national survey. J. Hosp. Med..

[8] Anesi GL (2022). Association of ICU admission and outcomes in sepsis and acute respiratory failure. Am. J. Respir. Crit. Care Med..

[9] Luo M, He Q (2023). Development of a prognostic nomogram for sepsis associated-acute respiratory failure patients on 30-day mortality in intensive care units: A retrospective cohort study. BMC Pulm. Med..

[10] Le Gall JR (2005). The use of severity scores in the intensive care unit. Intensive Care Med..

[11] Vincent JL (1996). The SOFA (Sepsis-related Organ Failure Assessment) score to describe organ dysfunction/failure. On behalf of the Working Group on Sepsis-Related Problems of the European Society of Intensive Care Medicine. Intensive Care Med..

[12] Singer M (2016). The Third International Consensus Definitions for Sepsis and Septic Shock (Sepsis-3). JAMA.

[13] Liu Z (2019). Prognostic accuracy of the serum lactate level, the SOFA score and the qSOFA score for mortality among adults with Sepsis. Scand. J. Trauma Resusc. Emerg. Med..

[14] Raith EP (2017). Prognostic accuracy of the SOFA score, SIRS criteria, and qSOFA score for in-hospital mortality among adults with suspected infection admitted to the intensive care unit. JAMA.

[15] Khwannimit B, Bhurayanontachai R, Vattanavanit V (2019). Comparison of the accuracy of three early warning scores with SOFA score for predicting mortality in adult sepsis and septic shock patients admitted to intensive care unit. Heart Lung.

[16] LeGall JR, Loirat P, Alpérovitch A (1986). APACHE II–a severity of disease classification system. Crit. Care Med..

[17] Ho KM (2006). A comparison of admission and worst 24-hour Acute Physiology and Chronic Health Evaluation II scores in predicting hospital mortality: A retrospective cohort study. Crit. Care.

[18] Le Gall JR, Lemeshow S, Saulnier F (1993). A new Simplified Acute Physiology Score (SAPS II) based on a European/North American multicenter study. JAMA.

[19] Soares M (2010). Validation of four prognostic scores in patients with cancer admitted to Brazilian intensive care units: Results from a prospective multicenter study. Intensive Care Med..

[20] Soares M, Salluh JI (2006). Validation of the SAPS 3 admission prognostic model in patients with cancer in need of intensive care. Intensive Care Med..

[21] Blanco J (2008). Incidence, organ dysfunction and mortality in severe sepsis: A Spanish multicentre study. Crit. Care.

[22] Poulose V (2008). Severe community-acquired pneumonia requiring intensive care: A study of 80 cases from Singapore. Singapore Med. J..

[23] Johnson AE, Kramer AA, Clifford GD (2013). A new severity of illness scale using a subset of acute physiology and chronic health evaluation data elements shows comparable predictive accuracy. Crit. Care Med..

[24] Chen Q, Zhang L, Ge S, He W, Zeng M (2019). Prognosis predictive value of the Oxford acute severity of illness score for sepsis: A retrospective cohort study. PeerJ.

[25] Jentzer JC (2020). Admission diagnosis and mortality risk prediction in a contemporary cardiac intensive care unit population. Am. Heart J..

[26] Johnson AE (2016). MIMIC-III, a freely accessible critical care database. Sci. Data.

[27] DeLong ER, DeLong DM, Clarke-Pearson DL (1988). Comparing the areas under two or more correlated receiver operating characteristic curves: A nonparametric approach. Biometrics.

[28] Vickers AJ (2008). Decision analysis for the evaluation of diagnostic tests, prediction models and molecular markers. Am. Stat..

[29] Vickers AJ, Van Calster B, Steyerberg EW (2016). Net benefit approaches to the evaluation of prediction models, molecular markers, and diagnostic tests. BMJ.

[30] Hotchkiss RS, Karl IE (1992). Reevaluation of the role of cellular hypoxia and bioenergetic failure in sepsis. JAMA.

[31] English M (1996). Deep breathing in children with severe malaria: Indicator of metabolic acidosis and poor outcome. Am. J. Trop. Med. Hyg..

[32] Kruse O, Grunnet N, Barfod C (2011). Blood lactate as a predictor for in-hospital mortality in patients admitted acutely to hospital: A systematic review. Scand. J. Trauma Resusc. Emerg. Med..

[33] Martin GS, Mannino DM, Moss M (2006). The effect of age on the development and outcome of adult sepsis. Crit. Care Med..

[34] Park SK (2009). Acute physiology and chronic health evaluation II and simplified acute physiology score II in predicting hospital mortality of neurosurgical intensive care unit patients. J. Korean Med. Sci..

[35] Al-Hadeedi S, Fan ST, Leaper D (1989). APACHE-II score for assessment and monitoring of acute pancreatitis. Lancet.

[36] Pollack MM, Patel KM, Ruttimann UE (1997). The Pediatric Risk of Mortality III–Acute Physiology Score (PRISM III-APS): A method of assessing physiologic instability for pediatric intensive care unit patients. J. Pediatr..

[37] Pérez-Fernández X (2017). Clinical variables associated with poor outcome from sepsis-associated acute kidney injury and the relationship with timing of initiation of renal replacement therapy. J. Crit. Care.

[38] Zhu Y, Zhang R, Ye X, Liu H, Wei J (2022). SAPS III is superior to SOFA for predicting 28-day mortality in sepsis patients based on Sepsis 3.0 criteria. Int. J. Infect. Dis..

[39] Le Gall JR (1996). The Logistic Organ Dysfunction system. A new way to assess organ dysfunction in the intensive care unit. ICU Scoring Group. JAMA.

[40] Vincent JL (1998). Use of the SOFA score to assess the incidence of organ dysfunction/failure in intensive care units: Results of a multicenter, prospective study. Working group on "sepsis-related problems" of the European Society of Intensive Care Medicine. Crit. Care Med..

[41] Ferreira FL, Bota DP, Bross A, Mélot C, Vincent JL (2001). Serial evaluation of the SOFA score to predict outcome in critically ill patients. JAMA.

[42] Tseng HY, Shen YC, Lin YS, Tu CY, Chen HJ (2020). Etiologies of delayed diagnosis and six-month outcome of patients with newly diagnosed advanced lung cancer with respiratory failure at initial presentation. Thorac. Cancer.

